# Supplementary material for: Reduction of aerosol dissemination in a dental area generated by high-speed and scaler ultrasonic devices employing the “Prime Protector”
Source: PLoS One. 2023 Aug 3;18(8):e0278791. doi: 10.1371/journal.pone.0278791 (PMC10399923; doi:10.1371/journal.pone.0278791)
Supplement: S1 File — (DOCX) [file pone.0278791.s001.docx]

**Reduction of aerosol dissemination in a dental area generated by high-speed and scaler ultrasonic devices employing the “Prime Protector”**

***Cleaning and disinfection protocol***

After the removal of the Petri dishes from the Preclinical laboratory, the authors were divided in three groups, in charge of the following cleaning and disinfection tasks:

**Group A:**

- Opening of the Preclinical laboratory’s windows
- Cleaning and disinfection of:
  - Hose and High-speed Handpiece (HH) and an Ultrasonic Prophylaxis Device (UPD):

1. Withdrawal and sealing of the tank containing the liquid suspension containing Lactobacillus casei Shirota.
2. Placement of a tank with purified water.
3. Activate the HH/UPD for 2 min.
4. Removal of the purified water container tank.
5. Placement of a ethyl alcohol (70%) container tank.
6. Activate the HH/UPD for 2 min.
7. Removal of the ethyl alcohol (70%) container tank.
8. Operation of the HH/UPD until observing dry air outlet.

**B Group:**

- Table cleaning:

1. Spray Benzalkonium chloride < 2% (Lysol) on the tables.
2. Rub in one direction (three times) with disposable absorbent paper towels.

- Floor cleaning (experimental area):

1. Dry any traces of moisture with a disposable absorbent paper towel.
2. Disinfection cleaning with disposable absorbent paper towels soaked in ethyl alcohol (70%).

**Group C:**

- Prime Protector device cleaning and disinfection:

1. Drying of possible traces of moisture from the stainless-steel base with a disposable absorbent paper towel.
2. Cleaning and disinfection of the stainless-steel base with disposable absorbent paper towels soaked in Benzalkonium chloride < 2% (Lysol).
3. Drying of possible traces of moisture from the dome with a disposable absorbent paper towel
4. Cleaning and disinfection of the dome with disposable absorbent paper towels soaked in Benzalkonium chloride < 2% (Lysol).
5. Closing of the Preclinical laboratory’s windows.
6. Control Petri dish placement.
7. Close the door.

**NOTE:**

**After finishing the experiment per day**

1. Unit feeder liquid container: wash with water and neutral liquid soap. Rinse with plenty of water.
2. Internal and external disinfection with ethyl alcohol (70%).
3. Let dry on rack.
