# Supplementary material for: Reduction of aerosol dissemination in a dental area generated by high-speed and scaler ultrasonic devices employing the “Prime Protector”
Source: PLoS One. 2023 Aug 3;18(8):e0278791. doi: 10.1371/journal.pone.0278791 (PMC10399923; doi:10.1371/journal.pone.0278791)
Supplement: Attachment — Submitted filename: Response to Reviewers.docx [file pone.0278791.s002.docx]

Dear Rajeev Singh,
Academic Editor
PLOS ONE

Based on the comments made by the academic reviewer, PLOS Editorial Office and reviewers, we proceed to answer each of the points.

For the PLOS Editorial Office. - We appreciate your recommendation to review and evaluate the specific previously published works and have taken them into account to improve our manuscript, as they are relevant to our investigation.

About the Journal Requirements. -

1. We have made sure that our revised manuscript meets PLOS ONE's style requirements.

2. We have requested the architect Lissette Lucio (author of Figure 1) for her written permission to publish these figures specifically under the CC BY 4.0 license, through the completion of the Content Permission and uploaded that file as an "Other" file with our submission.

To the reviewers, we considered it appropriate to resume the responses to their comments in the tables below.

To Reviewer #1: Laurence Walsh

We have considered your comments to our manuscript. The corrections change the lines’ numbers in the revised manuscript.

| Comment | Response |
| --- | --- |
| 1.For the abstract to be meaningful, it should state that there was no separate high-volume evacuation used, nor was there any air removal attached to the dome. It should also give the key dimensions of the dome such as its diameter and position in relation to the mouth. | The correction regarding evacuation used can be found in the new lines 38 and 39. The correction regarding key dimensions of the dome can be found in the new lines 30-32. |
| 2.On line 56, the accepted definition for aerosol particles is a size of less than 5 µm - not 50 µm. | The correction can be found in the new line 61. |
| 3.In the introduction on line 66, there is no mention of other effective risk reduction methods such as preprocedural use of antimicrobial mouth rinses. | The correction can be found in the new lines 75-78 |
| 4.In line 68 some discussion about the use of the dome would be useful. Without any connected air removal the dome will simply reduce some direct splatter, particularly back towards the faces of the dental staff, but given the well-known nature of dental aerosols there is no logical reason why these would be contained within a passive dome that has no air removal associated with it. | The correction can be found in the new lines 70- 74 |
| 5.On line 82, figure 1 part A needs to explain which of the simulator units within the simulation laboratory was used, and clearly indicate this in part a of the figure and also in the legends of that figure. | Figure 1 and its legend was corrected. |
| 6.On line 83 the description of the vertical and horizontal axes is confusing, since according to figure 1 part B these are behind the patient’s head and behind the operators head, however logically they should be towards the patient’s feet. | Figure 1 part B was corrected . |
| 7.For line 86, describe how the working services were disinfected. | Description can be found in lines 111-113. |
| 8.For line 104, state the nature of the insert used in the ultrasonic, and the type of bird that was used in the dental high-speed handpiece. Also specify what the water flow rate was to each of these devices (in mL/min). | Ultrasonic and dental high-speed handpiece bur type can be found in new lines 128 and 129.  Flow rate can be found in line 133 |
| 9.For line 113, a wider angle photograph of the prime protector should be included to show how the 3 feet are located in relation to the dental chair | Figure 2 was modified and a better description of the placement of the PP in lines 142-145 |
| 10.For line 124, based on the non-normal distribution of the counts, either a non-parametric method should have been used for all analyses, or the CFU data log transformed before analysis. This is relevant to the analysis of results in line 141 were a parametric test was used. | The correction can be found in the new lines 156-158. |
| 11.The legend to table 1 needs to are the key values - they are not meeting differences as is stated in the title of table 1. | Table 1 was modified. |
| 12.In the discussion on line 162, the authors should follow the hierarchy of risk controls, and list elimination, substitution, engineering controls, administrative controls and then PPE in that order from most effective to least effective. It is inappropriate to begin the discussion by listing barriers first. | We followed this recommendation, and the revised version is on lines 200-212 |
| 13.For line 166, several clinical studies have now shown that the combination of preprocedural mouth rinsing and the use of high-volume evacuation allows dental practice to be conducted safely. These recent studies should be cited at this point, see DOI 10.1177/00220345211015948; DOI 10.1007/s00784-022-04532-8; and similar papers. | We followed this recommendation, and the revised version is on lines 217-221 |
| 14.On line 201 it is stated that lactobacilli are Gram-negative - which is incorrect. While they could be described as facultative anaerobes it is technically more correct to describe them as being aerotolerant anaerobes. | The correction can be found in the new line 255 |
| 15.On line 219, the inclusion of high-volume evacuation should be mentioned, since this measure is used routinely in modern dental practice, and it is known to be highly effective at mitigating the generation of aerosols and the spread of splatter into the operatory. | The correction can be found in the new line 277 |
| 16.The authors could do a better job of discussing the limitations of their work, including the ways that their setup does not replicate what happens in a real working clinic. | The correction can be found in the new lines 270-274 |
| 17.On line 225 in the conclusion, the study did not test in any way the use of PPE so it is inappropriate to make commentary regarding the use of PPE.as well, PPE is the lowest of the risk control methods in terms of its hierarchy of use. | Usage of PPE was excluded from our conclusions as we didn’t test it |
| 18.Several typographical areas are present  a.line 124 the word no should be non  b.line 134 the word stablished should be established | The correction can be found in the new lines 156 and 161. |
| 19.The references require revision  a. to ensure correct format to match the journal (including removing the name of the editor from references 1 and 18,  and  b, to ensure the correct use of capitals in the title of articles in references 3, 6, 7, 8, 12, 13, and 22. | a. All editors from the references were eliminated. b. The titles of the mentioned articles were checked and corrected |
| 20.Reference 8 is missing details of the journal name, journal volume and year of publication. | Previous reference 8 is now reference 9, which has been corrected. |
| 21.The supplied link for sharing data does not work. | We have fixed this problem. Files can be found in the new link |
| 22.The legend to figure 1 should state what the bars represent (median, 95% CI etc). | Figure 1 did not have bars. Figure 3 had bars and its legend was corrected following this comment. |

To reviewer #2: Marcelo Henrique Napimoga

We have considered your comments to our manuscript. The corrections change the lines’ numbers in the revised manuscript.

| Comment | Response |
| --- | --- |
| 1-The introduction is somehow poor and disorganized, there are several different devices that was developed and tested during the most problematic phase of pandemics, what is the difference showed here? | The introduction was revised, and it changed all the manuscript’s line numbers. The difference between pre and post pandemic protective devices is established in line. We hope your concerns have been solved. |
| 2-In the Introduction the authors stated that the previous studies “recent studies with different procedures and methodologies show a variety of results that leave gaps in evidence” what gap are that? Which one this paper solved? Is not clear. Besides, there are similar devices as the author present, included also in the reference list Montalli et al., 2021; Chestsuttayangkul et al. | We have corrected the introduction and the contribution of our study is from line 79 to 88. |
| 3-One of the major concern to use a device like Prime Protector is the disinfection needed after procedures made in one patient. There is no information regarding this concern. Different devices like the one described by Montalli (<https://doi.org/> 10.1371/journal.pone.0255533) using a metal support, with a 30 cm ring and covered by a disposable 30 microns thickness PVC film measuring approximately 1.5 x 1.5 m which is disposable. How was the cleaning and disinfection made? The same PP was used more than once? How the authors assure the complete disinfection? If PP was used more than once during the experiment, is it possible to have contamination from the previous experiment? | Changes regarding the disinfection process can be found in lines 111-113 and the disinfection protocol is now uploaded as Supporting Information |
| 4-The methodology used should be referenced (<http://dx.doi.org/10.1590/1981-863720200001820200088>). | The correction can be found in the new lines 90-94 |
| 5-The table included in the text is not necessary since there is no relation with the manuscript. | The table mentioned was eliminated |
| 6-Discussion should be deeper revised. | We have added and edited the discussion following the recommendations from reviewer #1 |
| 7-The legend of the Figure 3 is not clear. | The legend of figure 3 was corrected |
| 8-There are some references with missing data in the list for example Chestsuttayangkul | The references were revised and updated. |

We hope to have met all the requirements and we are open to answering any other questions or concerns.

Thanks for your gentle attention.

Sincerely,

Dr. Carolina Guzmán

Correponding author
